# Nutritional and Environmental Factors in Thyroid Carcinogenesis

**DOI:** 10.3390/ijerph15081735

**Published:** 2018-08-13

**Authors:** Immacolata Cristina Nettore, Annamaria Colao, Paolo Emidio Macchia

**Affiliations:** Dipartimento di Medicina Clinica e Chirurgia, Università di Napoli Federico II, 80138 Napoli NA, Italy; immanettore85@libero.it (I.C.N.); colao@unina.it (A.C.)

**Keywords:** thyroid cancer, environment, carcinogens, iodine intake, eating habits, volcanoes, xenobiotics

## Abstract

Several epidemiological studies suggest an increased incidence of thyroid carcinoma (TC) in recent years, especially for the papillary histotype (PTC), suggesting that specific carcinogens might promote molecular abnormalities that are typical of PTC. The increased incidence is probably attributed to more intensive and sensitive diagnostic procedures, even if recent data suggest that various toxic elements could explain the phenomenon. Ionizing radiation exposure represents the most accepted risk factor for differentiated thyroid cancer that includes both the follicular and papillary histotypes. In this review, we examined the other environmental carcinogens that play a role in TC, such as eating habits, living in volcanic areas, and xenobiotic elements. Among eating habits, iodine intake represents one of the more discussed elements, because its deficiency is associated with follicular thyroid carcinomas (FTCs), while its progressive increment seems to be responsible for PTC. The gas, ash, and lava emissions of volcanoes are composed of various toxic compounds that pollute ground water, vegetables, and animals, contaminating humans via the food chain. Finally, the risk of developing PTC has also been associated with exposure of the population to xenobiotics in the environment or in the home. Their carcinogenic effects are probably caused by their accumulation, but additional studies are necessary to better understand the mechanisms of action.

## 1. Thyroid Cancer

### 1.1. Histotype and Clinical Features

Thyroid carcinoma (TC) is the most common endocrine malignancy and the cancer with the largest annual increase in incidence in the United States [1]. A recent study predicted that TC will become the third most common cancer in women by 2019, with a cost of $19–21 billion in the United States [2], and it has been estimated that it will be the fourth leading cancer diagnosis by 2030 [3]. Thyroid carcinoma occurs two to four times more frequently in females than in males. It is rare in children and adolescents, even if the risk of malignancy of thyroid nodules is major in young individuals [4]. 

The TCs can derive by either follicular thyroid cells or parafollicular cells (C-cells). According to their histopathological characteristics, follicular cell-derived carcinomas can be classified into: (i) papillary thyroid cancer (PTC; 75–85% of cases) characterized by excellent prognosis; (ii) follicular thyroid cancer (FTC; 10–20% of cases); (iii) Hürtle cell carcinomas (also known as oxyphilic cell carcinoma), rare and with prognosis similar to follicular carcinoma; (iv) anaplastic thyroid cancer (ATC), aggressive undifferentiated tumors with a disease-specific mortality approaching 100%; and (v) poorly differentiated thyroid cancer (PDTC), an uncommon form of thyroid carcinomas accounting for less than 5% of all cases [4,5].

Differentiated thyroid cancers comprise the vast majority (>90%) of all thyroid cancers [6], include papillary and follicular histotypes, and have a favorable prognosis according to the American Thyroid Association [4].

Medullary thyroid cancer (MTC) arises from the neuroendocrine parafollicular C cells of the thyroid. Sporadic MTC accounts for about 80% of all MTCs. The remaining cases consist of inherited tumor syndromes, including multiple endocrine neoplasia type 2A (MEN 2A) or multiple endocrine neoplasia type 2B (MEN 2B), and familial MTC [4,5]. 

### 1.2. Epidemiology

Several epidemiological studies concerning TC have suggested an increasing incidence of the tumor in the past three decades all over the world in adults, adolescents, and children [7,8]. 

Among the DTCs, the increased incidence regards mainly the PTC, in particular the follicular variant of PTC (FVPTC) [9]. The prevalence of follicular histotype showed a very modest increase [10], while the ATC remained constant or decreased [11]. These findings suggest that specific carcinogens might promote molecular abnormalities that are typical of papillary cancer.

The increased incidence of TC has been attributed to more intensive and sensitive diagnostic procedures [12,13], but it has also been suggested that improved diagnostic technologies may not fully explain the increased frequency of TCs [14]. Therefore, it has been proposed that environmental factors, lifestyle, and comorbidities could contribute to this phenomenon. 

Radiation exposure represents the most accepted risk factor for DTC, increasing the risk of thyroid malignancy from 5% to 50% [15], but several additional factors have been investigated, including cigarette smoking [16], estrogens [17], obesity [18,19,20], or diabetes [21]. In this review, we conducted a detailed examination of the additional elements playing a putative role in thyroid carcinogenesis, represented by eating habits, living in volcanic areas, and xenobiotic elements [22]. A summary of the data presented here is shown in Table 1.

## 2. Eating Habits

### 2.1. Iodine Intake

Iodine is an essential trace element for thyroid function, and it is necessary for human life. The introduction of iodine prophylaxis in a previously iodine-deficient population led to a reduction of FTC, but led to a predominant papillary histotype [36]. This supports the hypothesis that iodine deficiency is associated with an increased risk of FTC, whereas chronically high iodine intake may increase the risk of PTC [37] and more aggressive histological tumor features, such as lymph node metastases [38]. Iodine deficiency may lead to reduced thyroid hormone (T3 and T4) production and consequent hypersecretion of thyroid stimulating hormone (TSH). This induces hypertrophy and hyperplasia of thyroid follicular cells and promotes the onset of cancer [39]. 

The prevalence of *BRAF* (T1799A) mutation in a cohort of 1032 PTC patients from various regions of China with different degrees of iodine intake (from normal to high) was studied by Guan et al [23]. Data suggested that a high iodine intake could be a risk factor for *BRAF* (T1799A) mutation in the thyroid gland. Similar data have recently been obtained in a Polish institution [24]. In this study, the authors analyzed the frequency of *BRAF* (V600E) mutation, demonstrating the significant increase of mutation in PTCs patients. These data confirmed previous studies reporting that in iodine-replete areas the 97% of TCs were PTCs and that *BRAF* mutations were present in ≥80% of them [25,40].

In contrast, in a Korean population that chronically consumes iodine-rich foods, Kim et al. demonstrated that *BRAF* mutations in PTC were more frequent in subjects with either low (urinary iodine concentration, UIC < 300 µg/L) or excessive iodine intakes (UIC ≥ 500 µg/L) [26]. 

A detailed molecular analysis of PTCs and FTCs from an iodine-rich country (Japan) and an iodine-deficient country (Vietnam) was recently conducted by Voung et al. [36]. *BRAF* (V600E) mutation, *RET* rearrangements, and *RAS* mutations were analyzed, but the authors did not identify significant differences in genetic alterations in DTCs among the two examined regions, concluding that iodine intake did not influence the presence of mutations in patients with TC. 

Fish is considered an important source for iodine and other micronutrients, but it can contain also several contaminants that may potentially affect the thyroid or influence TC risk. The large prospective study EPIC (European Prospective Investigation into Cancer and Nutrition) performed in Europe, where both iodine deficiency and excess are rare, demonstrated that the consumption of fish or shellfish is not associated with changes in DTC risk [41].

### 2.2. Other Nutritional Influences

The association between total fruit and vegetable consumption and TCs was investigated by Zamora-Ros et al. using data from the EPIC cohort [42]. Fruit and vegetable intake is considered as probably protective against overall cancer risk, but the results of this study demonstrated that no association was present between TC and total fruit and vegetable intakes, vegetables, or fruit. However, a slight but positive trend of association was demonstrated between TC and fruit juice intake, probably correlated to their high sugar content [42].

In the EPIC Study, the association between alcohol consumption and DTC was also examined [43]. Contrarily to what has been suggested by other studies, reporting an increased risk of various forms of cancer with alcohol intake, this study suggested that individuals consuming 15 or more grams per day had a 24% lower risk of DTC compared to subjects consuming 0.1–4.9 g of alcohol. Separate analyses were performed by type of alcohol consumption (beer, wine, spirits), and data indicated that a reduced risk was demonstrated for TC in wine consumers, but not for beer or spirit consumers. Therefore, the mechanisms explaining the association between alcohol consumption and DTC risk are not clear and are potentially very complex.

In recent years, several studies have demonstrated an association between macronutrient ingestion and tumor susceptibility. The underlying mechanisms of these associations have not been completely clarified, but it has been suggested that carbohydrate consumption could promote insulin resistance and that protein intake is related to an increased risk of developing cancer, as consequence of the high nitrosamine content in some processed meat products. The relationships between macronutrient (carbohydrate and protein) consumption and DTC risk have recently been investigated [44]. This study demonstrated that in women, but not in men, a higher risk of DTC is associated with excessive caloric intake, excess of protein and carbohydrate, but not with high lipid and fiber intakes or physical activity [44].

A carbohydrate-rich diet is a potential risk factor for the development of insulin resistance (IR), and the impairment of insulin regulation might lead to a deregulation of the PI3K/AKT pathway, which has been strongly related to DTC development [45]. The data about excessive protein consumption is controversial. Several authors did not find any association between DTC risk and fish consumption [46], while intake of nitrite and nitrate through drinking water or food intake has been suggested to increase the risk of DTC in different studies [47,48]. The possible mechanisms by which nitrate may react is related to nitrate’s specific inhibition of iodide uptake by the thyroid. A decrease of the intra-thyroidal iodide can result in lower production of thyroid hormones and a consequent increase in thyroid stimulating hormone (TSH) levels. Chronic TSH stimulation of the thyroid is thought to be an important risk factor for thyroid carcinogenesis [47]. In addition, nitrate is converted to nitrite, which may react with amines and amides, promoting the formation of N-nitroso compounds (NOCs), known to be potent carcinogens [47] and associated with thyroid tumors in animal studies [49].

Finally, several studies have evaluated the relationships between nutritional vitamin D intake and cancers. Reduced vitamin D levels have been associated with an increased risk of several types of carcinomas, including TC, and with a major aggressiveness of thyroid cancers. Our group has recently reviewed data present in the literature studying the relationships between vitamin D levels and DTC, but no definitive conclusion can be drawn on either the association between low vitamin D levels and TC or on the possible effects of vitamin D supplementation on thyroid cancer risk [50].

## 3. Volcanic Area

### Studies Demonstrating the Higher Prevalence of Thyroid Carcinoma in Volcanic Areas

An increased risk of DTC has been reported in some volcanic areas of the world, such as Hawaii, Iceland, French Polynesia, New Caledonia, and Sicily [30,51].

The first association between thyroid cancer and active volcanoes was reported by Kung et al. [27], suggesting the presence of various carcinogenic agents in the lava of volcanoes in Iceland and Hawaii, regions where the incidence of thyroid cancer is remarkably high compared to other countries. The environmental factors act in combination with genetic factors and lifestyle, as possible causes of TC. The relationships between TC and volcano geography are such that the risk of developing TC were found to vary with birthplace [52]. Indeed, subjects belonging to the same ethnicity that were born in different geographical locations may have different risk of developing TCs, because of the different environmental conditions and the different concentrations of trace elements in their environment. These elements are present at higher concentrations in volcanic areas and may contaminate the soil and vegetables and affect the animal food chain [28]. 

Volcanoes are not all the same, and their gas, ash, and lava emissions are composed of various toxic compounds. 

Several studies have shown an increased content of heavy metals (iron Fe, chromium Cr, copper Cu, manganese Mn, nickel Ni, lead Pb, and zinc Zn, among others) in soil and plants grown in various volcanic areas, as well as in irrigation water. In addition, the emitted volcanic gases (CO_2_, sulfur, and chlorine compounds) are responsible for polluting the atmosphere [31,53]. These compounds also pollute ground water and, thereafter, vegetables grown in the area and animals (including humans) via the food chain. 

Heavy metals can act as carcinogens by causing genetic and epigenetic alterations in susceptible cells and favoring their malignant transformation. The sequence of events ranging from exposure to heavy metals (dose, duration, metal speciation) to the neoplastic transformation of the thyroid cells is still unknown, and the casual links between exposure to a carcinogenic metal and malignant thyroid transformation are not well established [54]. Moreover, it is very likely that is not the concentration of a single metal that causes the toxic or carcinogenic effect, but rather the synergistic effect of a complex mixture of interacting chemicals that induces organ damage, even at lower levels of exposure. 

In 2008, Frasca et al. suggested a possible association between thyroid carcinogenesis and a higher rate of *BRAF* mutation (V600E) in eastern Sicily (the location of the volcano Mount Etna), compared to western Sicily [55]. The highest radon levels were detected in the eastern sector of the island, which is the most seismically active zone [56]. Radon is a poisonous gas produced from the radioactive decay of uranium, and its concentration has been found to be increased in the ground water of the Mt. Etna area. 

The toxic effect of trace elements is mediated mostly via the aquiferous sources, and the role of vehicles in the transmission of biocontamination as well as in thyroid tumorigenesis has been underlined [54]. 

A recent retrospective study analyzed 500 patients with TC living in the area around the volcano Mt. Vesuvius (Campania, Italy). Parameters such as age, sex, tumor size, lymph-node invasion, distant metastases, cancer histotype, place of birth and of residence were investigated. The results confirmed the increase of PTC in subjects living in the volcanic area of Vesuvius than in nonvolcanic areas of the Campania region, suggesting a relationship between thyroid carcinoma and the volcano environment [29]. However, since Mt. Vesuvius has not been active for several years, the effect of its contamination should not be mediated via aquifer sources and therefore the concentration of volcanic mineral pollutants (i.e., selenium Se, vanadium V, and manganese) was similar between waters closer to the volcano and waters from nonvolcanic areas. 

By contrast, the large aquifer around the Mt. Etna, located in province of Catania, Italy, represents the main source of drinking water for about 700,000 residents of the area, and is rich in several heavy metals of volcanic origin. Contrary to Vesuvius, Mt. Etna is a continuously active basaltic volcano, and a variety of elements in the aquiferous sources around the volcano often exceed the maximum allowed concentration (MAC) fixed by the European and National regulations. Among these, essential elements such as iron and manganese are present, but also boron and vanadium, whose genotoxic and carcinogenic effects are unclear. Vanadium is considered as a possible human carcinogen influencing thyroid function and cell proliferation, and in vivo studies demonstrated its role in iodine metabolism and thyroid function by decreasing the thyroid peroxidase activity [57]. 

In the area surrounding Mt. Etna, the incidence of TC has been evaluated and data have been compared with those from nonvolcanic areas of Sicily. It has been demonstrated that the papillary histotype is more frequent than follicular or medullary carcinomas, and women were more commonly affected [30]. Subsequently a significant increase in PTC prevalence in pediatric age [32] has also been observed compared to a different area of Sicily.

Other potential thyroid disruptors present in waters from the area around Mt. Etna are fluorine, sulfur, and selenium. In general, the volcano’s rocks contain high levels of fluorine, which is transferred into groundwater. Mt. Etna is the largest known point source of atmospheric fluorine, but it has been largely demonstrated that excessive exposure to fluorine correlates to thyroid alterations [58].

Selenium is an essential micronutrient modulating several physiopathological processes in the thyroid gland [59]. Its anticancer role has been recently demonstrated [60], as well as the ability to act as antiapoptotic factor in thyroid follicular cells [61]. Its natural source is soil, plants, microorganisms, animals, and sea salt, but sulfur emitted by volcanoes competes with selenium compounds for uptake by plants, causing a decrease in available selenium [22]. As a constituent of selenoproteins, selenium has a structural and enzymatic role, so, its deficiency impairs thyroid function. Few studies have shown that selenium deficiency has been associated with an increased risk for several types of cancer, including thyroid cancer [62]. 

In conclusion, several studies have shown that living in a volcanic area could increase the risk of developing thyroid cancers. Millions of people are exposed to volcanic environments worldwide, and additional studies are necessary to identify specific environmental factors and thyroid carcinogens in these areas. These investigations will be helpful to understand the molecular mechanisms responsible for TC, and the development of preventive measures and major surveillance of exposed subjects.

## 4. Xenobiotics

The term “xenobiotics” refers to exogenous compounds and synthetic chemicals that can be released over time by construction and electronics materials, leading to diffuse exposure among the general population [63]. These compounds, also called endocrine-disrupting chemicals (EDCs), include flame retardants (FRs), pesticides, repellents, or thermal insulators—all products that improve our well-being but interfere with the biological functions and the homeostatic maintenance of the human organism. Recent studies evaluating the concentration in food, indoor and ambient air, and in-house dust have demonstrated that the levels of xenobiotic products differ in different parts of the world, and even within countries. 

Many of these compounds are not degradable, and therefore they accumulate in the environment and can be adsorbed by subjects through the food chain [64]. Diet is the main source of contamination for polybrominated diphenyl ethers (PBDEs) and hexabromocyclododecane (HBCD) during infancy in all countries, but especially in Germany and the UK [65]. In the United States, exposure to PBDE occurs mainly though incidental ingestion of indoor dust, inhalation of indoor air, and to a lesser extent, via dietary sources [64,66].

Recent data indicate that PBDEs exposure increased from the 1970s through to the early 2000s, but has recently decreased [67,68]. In the same direction were the results of a study conducted in California that compared PDBE levels in the serum of second-trimester pregnant women between 2008–2009 and a different cohort recruited in the same hospital in 2011–2012. Also this study demonstrated a a moderate but not significant decline in PBDE conventrations [69]. 

The reduction of PBDE concentrations in serum has been suggested to be determined by the more severe laws limiting the usage of such substances in recent years. By contrast, concentrations of different pollutants from fire retardants have recently been found increased when analyzing the dust in Californian homes [70]. In particular, higher concentrations of Firemaster^®^ 550 components were found in 2011 in comparison to 2006. 

Despite the reduction in the environmental concentrations of these products, it has to be noted that FRs may accumulate in several tissues and in breast milk [64], which may result in toxicity for several tissues, including thyroid. 

Moreover, these products remain ubiquitous in the environment because of bioaccumulation and biomagnifications, and therefore their concentrations will remain stable (or even increase) over time.

These products are responsible for thyroid toxicity via several mechanisms. The thyroid-specific effects of several industrial chemicals have recently been reviewed [71], and in a recent work their role has been evaluated in an in vivo system using zebrafish models [72]. 

One of these is related to their structural similarity to thyroxine, leading to alterations in circulating levels of hormones after exposure [73]. Polybrominated diphenyl ethers are able to inhibit the binding of thyroid hormones to transport proteins [74], and bind as agonist to the thyroid hormone receptors. Tetrabromobisphenol A (TBBPA) inhibits type 1 thyroid deiodinase activity, estrogen, and thyroid sulfotransferases [75]. Dioxins and PBDE decrease the half-life of T_4_ in serum by inducing the activity of hepatic uridine diphosphate glucuronyltransferases (UDPGTs), which glucuronidate T_4_ and determines its elimination via urines [76]. In 2008, Santini et al. suggested a possible interference of styrene with the peripheral metabolism of thyroid hormones by inhibiting the conversion of T_4_ to T_3_ [77]. All of these mechanisms could explain the reduced serum levels of T_4_ and the following increased levels of TSH in people exposed to chemical compounds.

Very recently, it has been demonstrated that higher levels of some flame retardants (BDE-209) were associated with increased risk (2.29 times) of PTC [33]. It is not clear how xenobiotics induce thyroid carcinogenesis, but a possible explanation could be the chronic hypersecretion of TSH and the autoimmune inflammatory reaction of the gland. These processes can induce the proliferation of thyroid follicular epithelium, favoring cell hypertrophy and hyperplasia, and secondarily thyroid oncogenesis [78]. Another explanation for the increased risk of PTC could be the presence of polymorphism involving xenobiotic-metabolizing genes [79,80]. The genetic polymorphisms are highly race-specific, and according to this finding, a recent work suggested the association between some specific single nucleotide polymorphisms (SNPs) and PTC risk in the Saudi Arabia population [79].

Since some of these compounds (e.g., PBDEs) are not metabolized by detoxification enzymes, the carcinogenic effects are probably caused by their accumulation. 

Some studies described a major risk of developing TCs for specific categories of workers. In Sweden, the occupational exposure of women in the shoe and leather industry to chemical solvents seems to be associated with increased TC [34]. In Shanghai, thyroid cancer was associated with a potential exposure to benzene, organic or inorganic gas, and formaldehyde [35]. 

## 5. Conclusions

This review described only some of the mechanisms that determine an increased risk of thyroid cancer as consequence of environmental factors. Data are consistent in animal models, but it is very difficult to demonstrate a clear cause–effect relation in human studies. Nevertheless, it is quite evident that the environment can modulate and influence not only the risk of thyroid cancer, but also the severity of the disease in humans. A long stay in xenobiotics-polluted or volcanic areas, low or high iodine intake, and some eating habits have been associated with TCs. Certainly, to better understand the mechanisms underlying the increased risk of developing thyroid cancers, additional studies are necessary in order to clarify the molecular mechanism causing the thyroid disruption, and especially, identify the specific elements to which human exposure can be restricted.

## Figures and Tables

**Table 1:** Environmental factors associated with increased risk of developing differentiated thyroid cancers (DTCs).

Risk Factor	Ethnicity	Number of Subjects	Year of Study	References
Iodine Intake	China	1032 PTC	2009	[23]
	Poland	723 PTC	2016	[24]
	Korea	n.a.	2018	[25,26]
Volcanic Area	Iceland and Hawaii	n.a.	1981	[27,28]
	Mount Vesuvius	500 TC	2012	[29]
	Mount Etna	1950 TC	2009	[30,31,32]
Xenobiotics Element	USA	n.a.	2017	[33]
	Sweden	n.a.	2009	[34]
	Shanghai	267,400 workers	2006	[35]

PTC: papillary thyroid cancer; TC: thyroid carcinoma; n.a: not applicable.
